# Sarcoidosis as a rare cause for symmetrical giant bullous disease

**DOI:** 10.1186/s12890-017-0429-z

**Published:** 2017-05-30

**Authors:** Wolfgang Jungraithmayr, Elisa Leggeri, Walter Weder, Bart Vrugt

**Affiliations:** 1 Department of Thoracic Surgery, University Hospital Zurich, Raemistrasse 100, 8091 Zurich, Switzerland; 2 Institute of Clinical Pathology, University Hospital Zurich, Zurich, Switzerland; 3 Department of Thoracic Surgery, Medical University Brandenburg, Campus Neuruppin, Germany

**Keywords:** Sarcoidosis, Lung, Giant bullous disease, Hypersensitivity pneumonitis

## Abstract

**Background:**

Sarcoidosis presents with typical clinic-radiological findings and shows histologically non-caseating granulomas. Pulmonary manifestations of sarcoidosis can be diverse, involving the intrathoracic lymph nodes and pulmonary parenchyma.

**Case presentation:**

We here describe a case of a 35-year-old patient who presented with a history of exertion dyspnoea and coughing for the past 20 years. At the age of 15, she was exposed to smoke emanating from a fire. Later, she had exposure to mold for two years, and during her childhood, she had animals such as a cockatiel, dog, cat, gecko, and turtle. Computed tomography of the chest revealed symmetrical apical giant bullous lesions. Histology of the resected bullae showed prominent peribronchial fibrosis with non-necrotizing, non-caseating granulomas and collaps of pulmonary lobules adjacent to the bulla. The absence of granulomatous infection and a markedly elevated CD4:CD8 ratio in bronchoalveolar lavage analysis suggested that the underlying process was sarcoidosis.

**Conclusion:**

In very rare cases, sarcoidosis can be associated with bilateral symmetrical apical giant bullous disease due to fibrotic and granulomatous changes resulting in a restriction of lung tissue.

## Background

Sarcoidosis is a granulomatous disorder of unknown aetiology and characteristically involves various organs, with lung involvement being predominant. The diagnosis of sarcoidosis is based upon the association of typical clinico -radiological findings and histological demonstration of non-caseating granulomas. The pulmonary manifestations of sarcoidosis are diverse, involving the intrathoracic lymph nodes and pulmonary parenchyma, as well as airways. However, pulmonary manifestations can be atypical. One such atypical manifestation can be a bullous form of the disorder. We here describe a very rare manifestation of pulmonary sarcoidosis showing bilateral, symmetrical, apical giant bullae.

## Case presentation

**Fig. 1:**
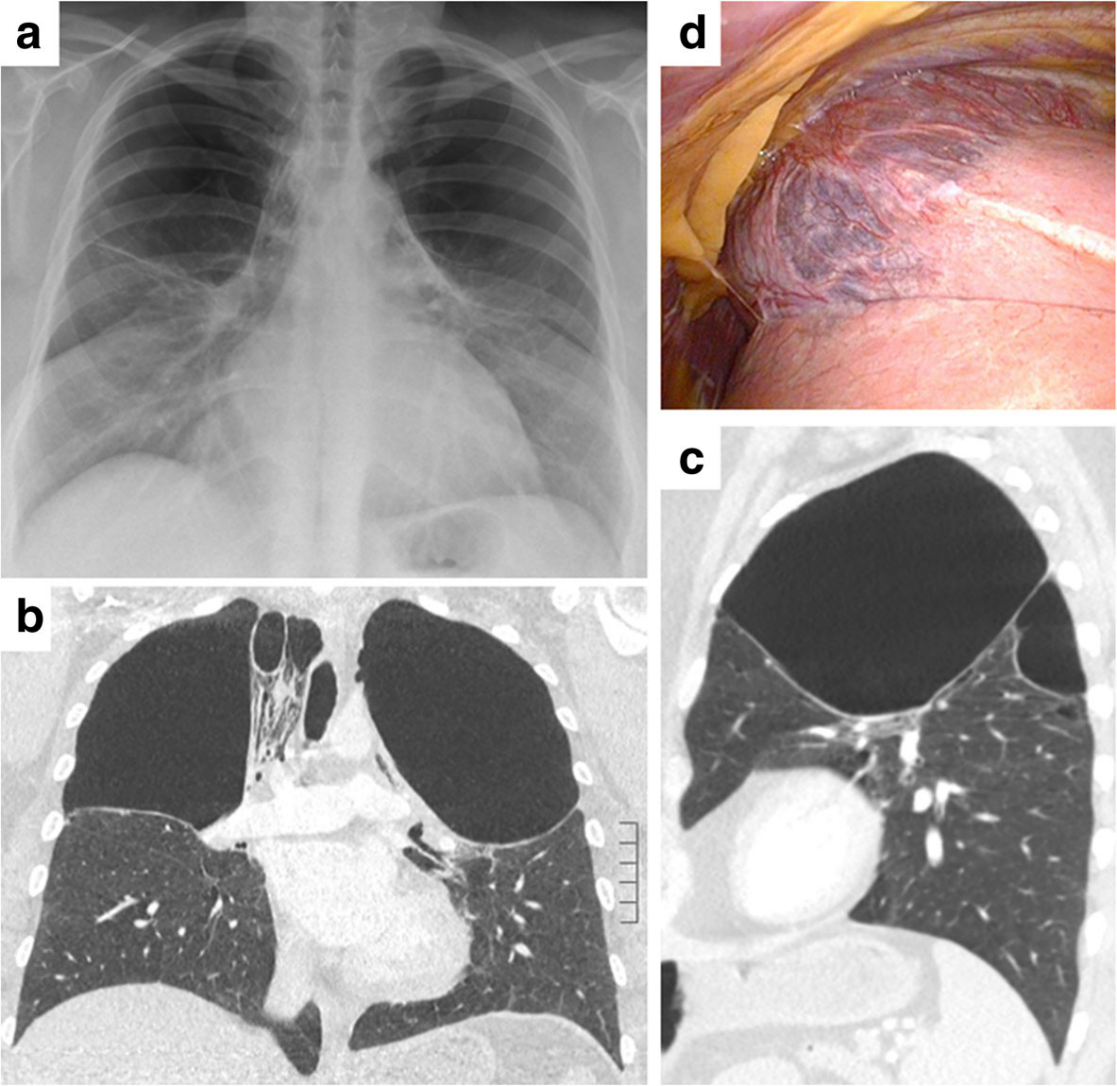
X-ray and computed tomography of the chest revealing large and symmetrical apical bullae (**a-c**). Intraoperatively, these bullae could be confirmed on the *right side* to originate mainly from the *upper* lung lobe (**d**)

A 35-year-old female patient presented with a history of exertion dyspnoea and coughing for the past 20 years. This lifetime non-smoker was otherwise healthy with no prior history of lung disease. On two separate occasions at the age of 15, she was exposed to smoke emanating from a fire. Later, at the age of 23, she was exposed to mold for two years. During her childhood, the patient had animals such as a cockatiel, dog, cat, gecko, and turtle. An actual x-ray and a computed tomography (CT) of the chest revealed symmetrical apical giant bullous lesions (Fig. 1a-d). Lung function analysis (percent predicted, post dilatation) revealed a FEV1 of 52%, a DLCO of 50%, and the FVC was 74%. α1-Antitrypsin deficiency was ruled out, and levels of IgE were found to be within normal ranges. Serum titers against red bloodworm, duck and goose feather were negative.

Intraoperatively, large apical bullae were found while the remaining lung showed a normal appearance and texture (Fig. 1d). To improve respiratory function, thoracoscopic bullectomy of the right side was performed. Upon discharge, the patient experienced an improvement of her respiratory condition and less coughing.

**Fig. 2:**
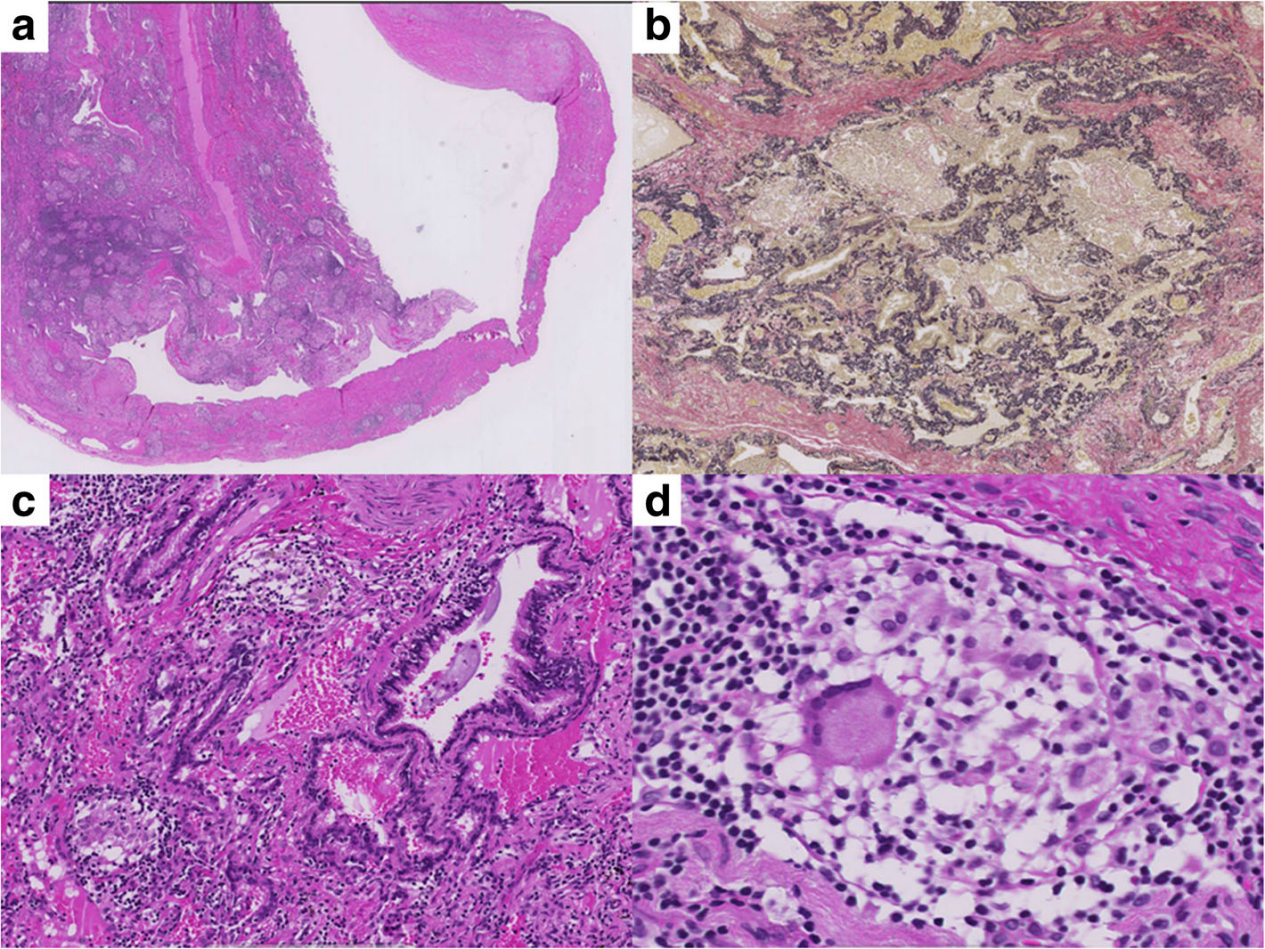
Low magnification H&E histology illustrates the transition from lung tissue to bulla (**a**) (magnification 25x). The peripheral lung tissue shows a fibrotic collaps of the secondary pulmonary lobules (**b**, EvG stain, magnification 40x). At higher magnification, a prominent peribronchiolar fibrosis as well as small granulomas with giant cells (**c**, magnification 40x, and **d**, magnification 60x) can be appreciated

Histological work-up showed prominent peribronchial fibrosis with numerous non-necrotizising, non-caseating granulomas and a collaps of pulmonary lobules adjacent to the bulla was found. Based on the histological findings, sarcoidosis was suspected (Fig. 2a, b) with hypersensitivity pneumonitis (HSP) being the main differential diagnosis.

## Discussion and conclusions

Sarcoidosis presents radiologically in the vast majority with thoracic lymphadenopathy, potentially accompagnied by lung parenchymal alterations such as pulmonary reticulo-nodular changes. The association of sarcoidosis and the presence of large bullae, however, is rare [1–3]. Although we did not find hilar lymphadenopathy nor sarcoidosis-related changes in the rest of the patients lung, the presence of non-caseating and non-necrotizing granulomas that were found adjacent to the bullae suggests a localized manifestation of pulmonary sarcoidosis. Fibrotic and granulomatous changes on the base of sarcoidosis most likely resulted in a restriction of lung tissue and in the development of the bullae. An enhanced CD4/CD8 lymphocyte ratio found in the bronchioalveolar lavage (8.8) further supported the diagnosis of sarcoidosis as the underlying causative disease. In addition, the history of fire exposures the patient suffered at a young age could be significant. These episodes were described as short and intense. This could have led to damage of the small respiratory bronchioles, which could potentiate the effect of a second insult (i.e. sarcoidosis) on the development of bullae, especially in the upper lung.

The main differential diagnosis to our case presented here is HSP. This pulmonary disease induces lung inflammation also showing non-caseating, non-necrotizising granulomas that result from inhalation of an allergen implicating previous sensitization [4]. Radiologic imaging can show centrilobular nodules, multifocal ground glass opacities, and evidence of air trapping in the expiratory phase of respiration, however, bullae are usually not seen in HSP. One case is known in a patient with farmers lung which showed a bulla in the context of HSP [5]. However, the bulla described was unilateral in contrast to the bilateral presentation in our patient.

Another cause for the development of these parenchymal alterations is mycobacterial infection. However, we could not detect mycobacterial complexes or atypical mycobacteria such as mycobacterium bovis, avium or scrofulaceum in the PCR analysis.

Taken together, the combination of fibrotic and granulomatous, non-caseating changes together with a high CD4:CD8 ratio in BAL favour a chronic form of sarcoidosis as the most likely cause for the development of giant symmetric lung bullae.

## Abbreviations

CT: Computed tomography
DLCO: Diffusing capacity or Transfer factor of the lung for carbon monoxide
FEV1: Forced expiratory volume in 1 s
FVC: Forced expiratory vital capacity
HSP: Hypersensitivity pneumonitis
PCR: Polymerase chain reaction
